# FAM19A4 and hsa-miR124-2 Double Methylation as Screening for ASC-H- and CIN1 HPV-Positive Women

**DOI:** 10.3390/pathogens13040312

**Published:** 2024-04-11

**Authors:** Cinzia Peronace, Erika Cione, Diana Marisol Abrego-Guandique, Marco De Fazio, Giuseppina Panduri, Maria Cristina Caroleo, Roberto Cannataro, Pasquale Minchella

**Affiliations:** 1 Unit of Microbiology and Virology, PO Pugliese, AOU Renato Dulbecco, 88100 Catanzaro, Italy; marco_2592@yahoo.it (M.D.F.); pandurigiuseppina@gmail.com (G.P.); pminchella@aocz.it (P.M.); 2 Department of Pharmacy, Health and Nutritional Sciences, University of Calabria, 87036 Rende (CS), Italy; 3 Galascreen Laboratories, University of Calabria, 87036 Rende (CS), Italy; dianamarisol.abregoguandique@unicz.it (D.M.A.-G.); mariacristina.caroleo@unicz.it (M.C.C.); r.cannataro@gmail.com (R.C.); 4 Department of Health Sciences, University of Magna Graecia, 88100 Catanzaro, Italy

**Keywords:** ASC-H, CIN1, double methylation, microRNAs

## Abstract

The DNA methylation levels of host cell genes increase with the severity of the cervical intraepithelial neoplasia (CIN) grade and are very high in cervical cancer. Our study aims to evaluate FAM19A4 and hsa-miR124-2 methylation in Atypical Squamous cells with high-grade squamous intraepithelial lesions (ASC-H) and in CIN1, defined as low-grade squamous intraepithelial lesions (LSILs) by the Bethesda classification, as possible early warning biomarkers for managing women with high-risk HPV infections (hrHPV). FAM19A4 and hsa-miR124-2 methylation tests were conducted on fifty-six cervical screening samples from a subset of women aged 30–64 years old. Specimens were collected into ThinPrep PreservCyt Solution. Their HrHPV genotype and cytology diagnosis were known. A Qiasure (Qiagen) was used for FAM19A4 and hsa-miR124-2 methylation testing on bisulfite-converted DNA, according to the manufacturer’s specifications. The reported results were hypermethylation-positive or -negative. We found that FAM194A4 and hsa-miR124-2 methylation was detected in 75% of ASC-H cases with a persistent infection of hrHPV. A total of 60% of CIN1 lesions were found to be positive for methylation, and 83.3% were when the cytology was CIN2/3. In addition, as a novelty of this pilot study, we found that combined FAM19A4 and hsa-miR124-2 methylation positivity rates (both methylated) were associated with the HPV genotypes 16, 18, and 59 and covered 22 and 25% of ASC-H and CIN1 cases, respectively. The methylation of these two genes, in combination with HPV genotyping, can be used as an early warning biomarker in the management and follow-up of women with ASC-H and CIN1 to avoid their progression to cervical cancer.

## 1. Introduction

**Figure 1:**
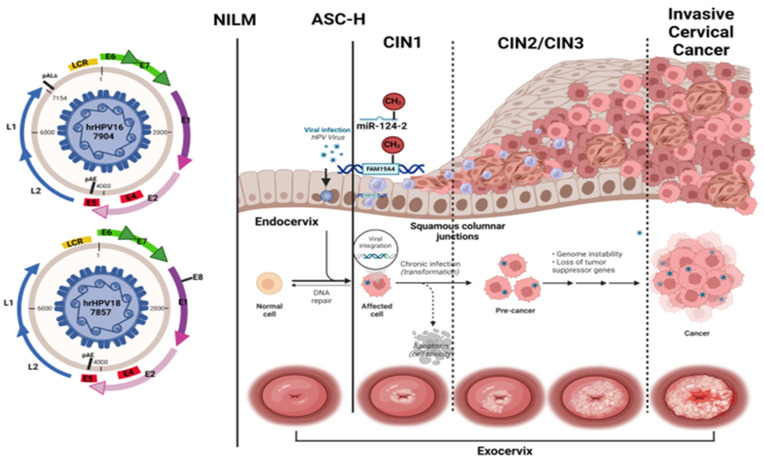
Methylation into ASC-H and CIN1 in hrHPV infections. Other lesions include CIN2/3 and Cervical Squamous Cell Carcinoma and Endocervical Adenocarcinoma (CESC).

The epidemiological surveillance of human papillomavirus (HPV) infection and its related diseases is crucial for monitoring and evaluating the currently available antiviral prophylactic vaccines [1]. HPV infection is the primary cause of cervical cancer among women [2]. HPV infection positivity occurs in more than 80% of cervical cancer cases worldwide [3]. Among women with normal cervical cytology, the highest HPV prevalences were found in Oceania (21.8%, estimated to be 30.9% in 2019) and Africa (21.1%), followed by Europe (14.2%), America (11.5%), and Asia (9.4%) [4]. In addition, HPV infection rates are higher in developing regions (42.2%) than in developed regions (22.6%) [5,6,7]. Nevertheless, its prevalence is quite high in Eastern Europe (21.4%). Adolescent girls and women under 25 were the most infected. However, in the African (East and West Africa) and American (Central and Southern America) regions, there was a rebound in HPV infections in adults over 45 years old [4,8]. In Italy, HPV infection data emphasizes the importance of the 9-valent vaccine as well as their screening program for cervical cancer; 2918 new cases of cervical cancer show the burden of disease attributable to HPV in Italy, where there are 2065 cases of neck cancer in both genders and about 100 new cases of penile cancer per year in men [9]. HPV infections are associated with cervical intraepithelial neoplasia (CIN), which can be divided into low- and high-scale risk grades. A low-grade CIN1 lesion can spontaneously resolve; in fact, it is also referred to as low-grade squamous intraepithelial lesions (LSILs). CIN2 refers to the abnormal changes in the epithelial cervical layer as a grey zone, since 50% of these lesions can regress, especially in young women [10]. The last grade is CIN3, or high-grade squamous intraepithelial lesions (HSILs), which is the most severe form, for which surgical treatment is needed. Over the past few years, it has become evident that epigenetic events, and in particular differential HPV gene methylation events, substantially contribute to the regulation of the papillomavirus’s life cycle [11,12] and, therefore, to infection progression. It is well-recognized and robustly proven that silencing tumor suppressor genes through the local hypermethylation of CpG-rich promoter regions contributes to cancer development [13,14]. DNA methylation, in the 5′ position of a cytosine molecule in CpG dinucleotides [15], is a biochemical mechanism that induces the covalent binding of a methyl group to this region. Methylation analysis is a promising triage tool for high-risk HPV (hrHPV)-positive women [16]. Studies have shown that the DNA methylation levels of host cell genes increase with the severity of the CIN grade and are very high in cervical cancer [17,18]. The functional relevance of methylation-mediated gene silencing during HPV-induced carcinogenesis in the host has been demonstrated for a subset of the currently known methylation gene targets, including CADM1, MAL, PAX1, FAM19A4, and hsa-miR124-2 [18,19]. It is worth mentioning that the absence of both FAM19A4 and hsa-miR124-2 methylation is associated with the high regression rate of CIN2 lesions, as was reported in the CONCERVE Study [20], which also highlights the regression in Atypical Squamous cells with high-grade squamous intraepithelial lesions (ASC-H) when negative for methylation. CIN1 and ASC-H represent a clinical dilemma since a variable percentage, from 5% to 20%, of this type of lesion progresses to HSILs and cancer. Our aim in this study was to evaluate both the FAM19A4 and hsa-miR124-2 methylation grades, in combination and alone, in the Atypical Squamous cells of a cytology of Undetermined Significance (ASC-H) and CIN1, also defined low-grade squamous intraepithelial lesions (LSILs), of women with high-risk HPV infections—with respect to the fact that CIN2/3 is also defined as high-grade squamous intraepithelial lesions (HSILs) by the Bethesda classification [21]—as well as in negative samples, as we speculate that it might be double FAM19A4 and hsa-miR124-2 methylation women that progress to cervical cancer (Figure 1).

## 2. Materials and Methods

**Scheme 1:**
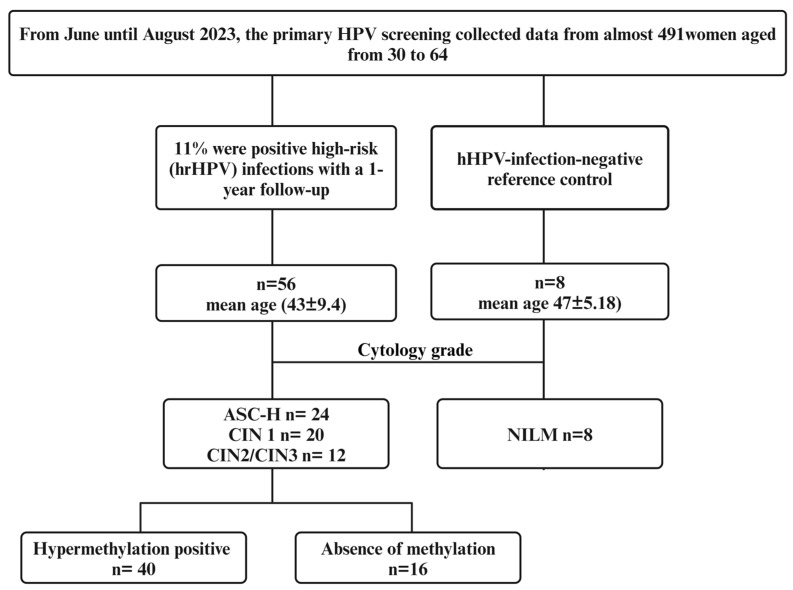
Flowchart of HPV screening.

From June until August 2023, in the central area of Catanzaro in the Calabria region, following a primary HPV screening, we collected data from 491 women aged from 30 to 64; 11% were at their one-year follow-up. Specimens were collected into ThinPrep PreservCyt Solution (Hologic, Marlborough, MA, USA). An explicative flowchart is in Scheme 1. Data were analyzed retrospectively. Multiplex real-time PCR utilizing Dual Priming Oligonucleotide (DPO) and Tagging Oligonucleotide Cleavage and Extension (TOCE) technologies (Anyplex II HPV HR Detection Seegene, distributed in Italy by Arrow Diagnostics) were used to detect 14 high-risk HPV genotypes simultaneously in a single reaction tube. A Qiasure (Qiagen) was used for FAM19A4 and hsa-miR124-2 methylation testing on bisulfite-converted DNA, according to the manufacturer’s specifications. Based on a three-step reaction, this technique involves treating methylated DNA with bisulfite, which converts unmethylated cytosines into uracil. Methylated cytosines remain unchanged during this treatment. Once converted, the methylation profile of the DNA can be determined by PCR amplification (Zymo Research, Irvine, CA, USA) using an input sample of 2.5 µL of bisulfite-converted DNA and the PCR instrument. Additional quality assurance was employed using the housekeeping gene β-actin (ACTB) as a reference for successful bisulfite conversion, sample quality, and signal normalization. According to the manufacturer’s instructions, the software runs the assays, followed by automatic quality assurance and data analysis, resulting in ΔCt and ΔΔCt value thresholds for FAM19A4 and/or hsa-miR124-2. Briefly, the ΔCt values were calculated as the difference between the Ct value of the FAM19A4 or hsa-miR124-2 targets and the Ct value of the reference (ACTB). For normalization, the ΔCt value of a calibrator sample (standardized low-copy plasmid DNA sample) is subtracted from the ΔCt of the FAM19A4 or hsa-miR124-2 targets, resulting in a ΔΔCt value. The reported results were methylation-positive for FAM19A4 and -negative for hsa-miR124-2, methylation-negative for FAM19A4 and -positive for hsa-miR124-2, or methylation-positive for FAM19A4 and -positive for hsa-miR124-2 (see Supplementary Materials).

### Bioinformatics

We used bioinformatics tools, the MethHC version 2.0 platform released in 2021, for assessing methylation levels and the Kyoto Encyclopedia of Genes and Genomes (KEGG) to identify the biochemical pathways related to FAM19A4 and hsa-miR124-2.

## 3. Results

Using a retrospective approach, we included a subset of cervical samples with a 1-year follow-up within the screened cohort, resulting in a percentage (11%) of women with positive hrHPV infections and a median age 43 ± 9.4 (range: 30–64 years) being included in the study. As a control reference, we included in the trials eight samples negative for intraepithelial lesion malignancies (NILMs) and negative for hrHPV DNA (mean age 37 ± 5.18), as shown in Table 1. Twenty-four women with ASC-H (mean age 42 ± 8.96) and with a higher prevalence of the hrHPV genotypes 16, 18, and 31, highlighted in bold, had three coinfections. Twenty were diagnosed with CIN1 (mean age 40 ± 8.25) and a higher prevalence of the hrHPV genotypes 18, 33, and 56, with one coinfection, and twelve with CIN2/3 (mean age 49 ± 10.09), a higher prevalence of the hrHPV genotypes 16, 39, and 59, and two coinfections, as highlighted in bold. The median time between the baseline clinically collected sample and baseline cytology was 28 days. Table 1 shows the methylation positivity rates, stratified by cytology grade and hrHPV positivity. We found that FAM194A4 and hsa-miR124-2 methylation detect the CIN2/3 lesions at the highest risk of progression to cervical cancer and, with a long-lasting HPV infection, these increase with age.

Concerning ASC-H, 75% of cases were recognized as positive for methylation, with a persistent infection of hrHPV. Of the CIN1 lesions, 60% were found to be positive for methylation in the methylation analysis, as shown in Table 2. The FAM19A4 and/or hsa-miR124-2 methylation tests were highest, equal to 83.3%, when the cytology was CIN2/3. The hrHPV genotypes for the cytology grades are also reported in the note regarding the asterisks.

**Figure 2:**
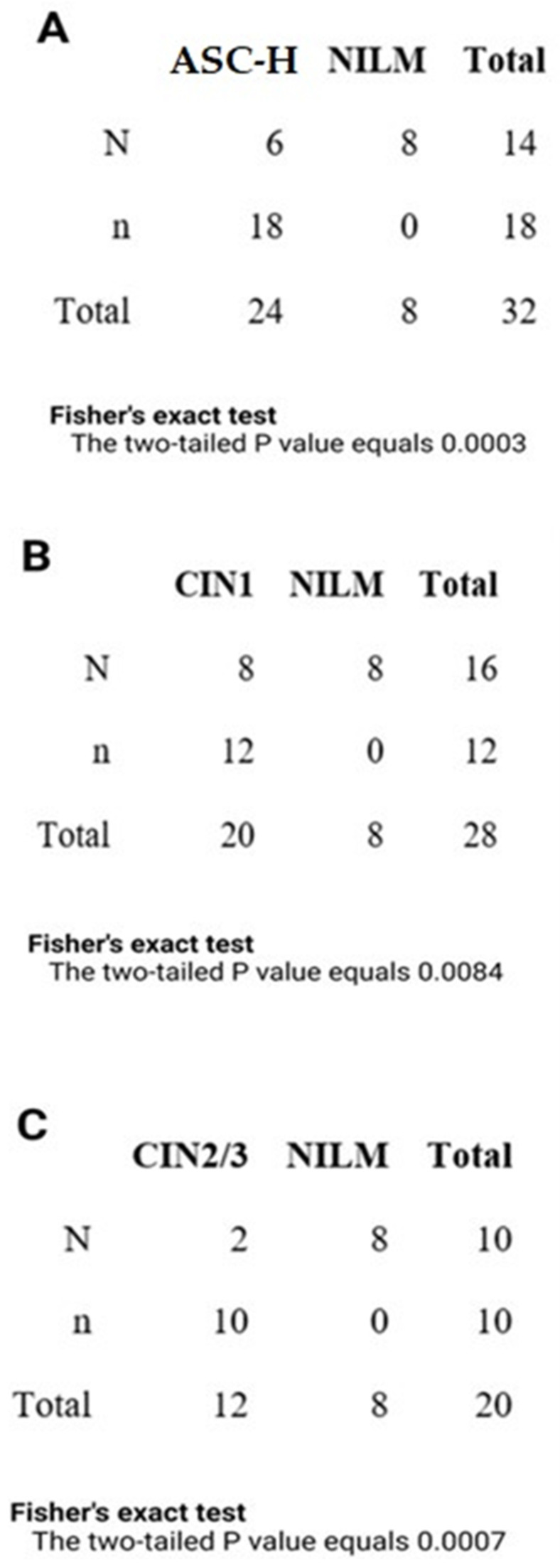
Fisher’s exact tests. (**A**) ASC-H versus NILM; (**B**) CIN1 versus NILM; and (**C**) CIN2/3 versus NILM. N = group total, n = number of positive FAM19A4 and/or hsa-miR124-2 methylations.

For the small sample size analyzed here, Fisher’s exact test was conducted to create contingency tables. The data show that the association between ASC-H and NILMs and methylated gene groups is considered to be extremely statistically significant, with a two-tailed *p*-value of 0.0003; it is very statistically significant between CIN1 and NILM, with a two-tailed *p*-value of 0.0084; and between NILM and CIN2/3 methylated genes it is extremely statistically significant, with a *p*-value of 0.0007, as is shown in Figure 2, in panels A, B, and C, respectively.

Table 3 shows the FAM19A4 and hsa-miR124-2 methylation results, in combination and alone, linked to the hrHPV genotyping positivity rates. We found that the combined FAM19A4 and hsa-miR124-2 methylation positivity rate was associated with the HPV genotypes 16, 18, and 59, as is highlighted in bold. In addition, we found that the positivity rate of FAM19A4 methylation alone was associated with the HPV genotypes 16, 18, 39, and 66, as highlighted in bold, and the positivity rate of hsa-miR124-2 methylation alone was associated with the HPV genotypes 16, 18, 31, 39, and 58, as highlighted in bold.

Table 4 shows the FAM19A4 and has-miR124-2 methylation results in combination and alone. We found that the methylation of both the FAM19A4 and hsa-miR124-2 genes in ASC-H was 22.2%, while in CIN1 it was 25%, and in CIN2/3 20%. In addition, the methylation positivity results for FAM19A4 alone show the following percentage values: ASC-H, 33.4%; CIN1, 50%; and CIN2/3, 50%. Those of hsa-miR124-2 show the following percentage values: ASC-H, 44.4%; CIN1, 25%; and CIN2/3, 30%.

**Figure 3:**
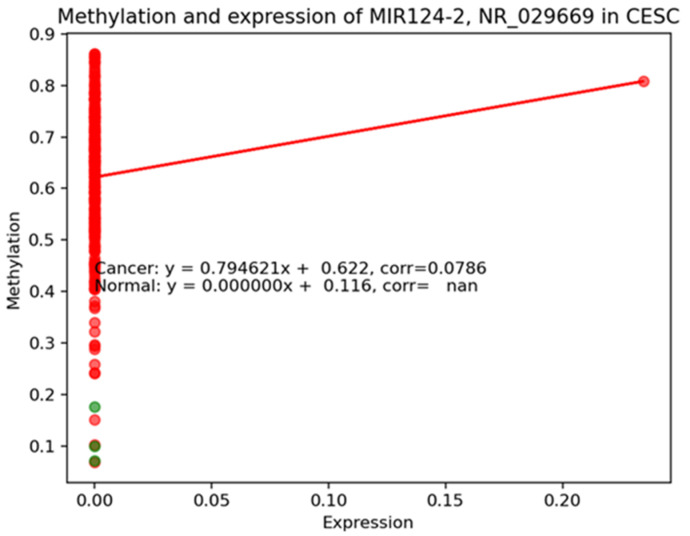
Methylation and expression of hsa-miR124-2 in CESC.

The methylation testing of Cervical Squamous Cell Carcinoma and Endocervical Adenocarcinoma (CESC)’s FAM19A4 and hsa-miR124-2 was conducted using the MethHC version 2.0 platform, released in 2021, which has counted 27,190 DNA methylation data points, 1732 expression data points, and 11,196 microRNA data points in 33 different types of tumors. The data on the methylation and expression of hsa-miR124-2 in CESC are shown in Figure 3; the plot shows its methylation on the *y* axis and its expression on the *x* axis. The level of hsa-miR-124-2’s expression in normal tissue is the baseline, and it increases up to 0.2 in CECS. The KEGG analysis of hsa-miR-124-2, which highlighted its importance in cancer pathways, is present in the Supplementary Materials (Figure S1). No data are reported for FAM19A4, even when searching for it in the MethHC version 2.0 platform using the alias TATA4, or in KEGG.

## 4. Discussion

Human health and disease are not only maintained by the DNA code, but also by the precise regulation of gene transcriptions and their epigenetic biochemical regulatory apparatus. Non-coding RNAs, either long or microRNA, are also part of this regulatory mechanism, in which the methylation patterns in normal cell physiology are often disturbed by aberrant cell growth or viral infections [22]. DNA methylation is the most extensively studied epigenetic change in HPV-related cancers. Although cervical cytology, also known as Pap test screening, is an effective tool for identifying premalignant changes in the cervical epithelium, a clinical debate is still ongoing with respect to the management of low-grade cervical abnormalities, known as ASC-H and CIN1. In this study, using a retrospective approach, we analyzed the methylation of FAM19A4 and hsa-miR124-2 in a subset of cervical samples of persistent HPV infections. A similar study was conducted in a large cohort of hrHPV specimens from several European countries. In any case, in that study, data from Italy were not present [23], and the double methylation of FAM19A4 and has-miR124-2 was not reported, as we pointed out here (see Table 3). The characteristics of the clinical patients in our subset of samples with positive hrHPV infections are in line with the literature. Indeed, globally, the most common HPV viral genotypes infecting women with a normal cytology are HPV 16, 31, 52, and 53. Similar data were found in a retrospective study about the genotype distributions in our region, with a prevalence of 16.9% for HPV 16, followed by 9.1% for HPV 31 [24]. In cervical cancer, the major viral genotypes are HPV 16 and 18, with over 55% and 14% of cervical cancers associated with each, respectively [4]. The overall mean age of the women was 43 ± 9.4. The ASC-H group had a mean age of 42 ± 8.96, with the occurrence of the hrHPV genotypes 16/18/31. The CIN1 group had a mean age of 40 ± 8.25 and was positive for the occurrence of the genotypes hrHPV 18 and 33, while the CIN2/3 group had a mean age of 49 ± 10.09 and was positive for the occurrence of the hrHPV genotypes 16, 39, and 59. The methylation of FAM194A4 and hsa-miR124-2 was highlighted in our results. In particular, the total methylation percentage found in ASC-H was 75%, while it was 60% for CIN1. The methylation was higher in CIN2/3, equal to 83.3%, as expected since DNA methylation is a well-established method for regulating gene expression. In ASC-H, methylation was already evidenced in the PAX1 gene [25] and other methylation markers such as ASCL1, LHX8, ST6GALNAC5, GHSR, ZIC1, and SST were found in CIN1. To assess their significance Fisher’s exact test was conducted. The contingency table shows that, for ASC-H and NILM, these methylated genes are considered to be extremely significant, as their difference had a two-tailed *p*-value of 0.0003; the findings from our study support the need for regular cytological follow-ups of women with ASC-H, as recommended so far by the American Society for Colposcopy and Cervical Pathology’s 2006 Consensus Guidelines [26]. The difference in CIN1’s methylated genes compared NILM’s has a two-tailed *p*-value of 0.0084. As expected, CIN2/3 evidences the same statistical trend, with a *p*-value of 0.0007. The methylation of hsa-miR124-2 is linked to Cervical Squamous Cell Carcinoma and Endocervical Adenocarcinoma (CESC). In our subset of samples, we found that hsa-miR124-2 shows the following positive percentage values: in ASC-H, 44.4%; in CIN1, 25%; and in CIN2/3, 30%. The methylation rate of both the hsa-miR-124-2 gene and the FAM19A4 gene (double methylation) in ASC-H was 22.2%, in CIN1 it was 25%, and in CIN2/3 20%. In addition, FAM19A4 methylation positivity alone shows the following percentage values: ASC-H, 33.4%; CIN1, 50%; and CIN2/3, 50%, respectively. It is important to remember that CIN2/3 lesions with an HPV infection have the highest risk of progression to cervical cancer and that this increases with age. Dovnik and Poljak also reported these results in a recent review. The authors showed that DNA methylation accurately predicts disease progression and is a valid triage tool for HPV-positive women, with CIN2 performing better than triage cytology [27]. A negative methylation result for both FAM19A4 and hsa-miR-124-2 was associated with a low long-term risk of cervical cancer in a Dutch longitudinal study [28]. We found that the genotypes 16 and 18 were frequently associated with FAM19A4 and/or hsa-miR-124-2 (even both) methylation positivity results in hrHPV infections.

In our opinion, this class of double positive results for FAM19A4 and hsa-miR-124-2 methylation should be considered more closely, by applying an extended genotyping screening test instead of a partial (only 16 and 18 or others). Methylation tests such as FAM19A4 and hsa-miR-124-2, in combination with cytology or HPV genotyping, can be used as an early warning biomarker in the management of women with CIN1 or ASC-H. Both represent a clinical dilemma in terms of their management, since from 5.2% to 18.8% of these types of lesion progress to HSILs and cancer [29,30]. The test is proposed as a substitute or addition to cytology for the reflex testing of HPV in screen-positive women. The worldwide prevalence of cervical HPV infections is estimated to be 11.7% (95% CI: 11.6–11.7%) in women with normal cytological findings and is significantly higher for women with an abnormal cytology even after surgery and vaccination [31,32,33]. The advent of FDA-approved molecular testing for diagnosing HPV infections has led to a dramatic shift from cytology testing alone to a combination of cytology and molecular testing for primary HPV screening. This screening practice should also be considered for the gene methylation of hrHPV, as was recently pointed out by Salsa and co-workers [34]. In fact, in their recent systematic review and meta-analysis, it was confirmed that DNA methylation-based markers constitute a promising tool for assessing hrHPV positivity and decreasing its overdiagnosis and overtreatment. With this type of screening program, the pressure on colposcopy units could decrease, improving waiting times and health costs. The advancement of cervical cancer screening programs over the decades has been constant, searching for the optimal balance between reliability and effectiveness. Methylation markers may be the next advancement, improving women’s adhesion to their screening, cost-effectiveness, and improving women’s quality of life. In line with these conclusions and pioneering this vision, in July 2019, the governments of the Netherlands and Turkey were the only in Europe to implement national HPV-based cervical cancer screening fully. Sweden, Finland, and Italy have implemented HPV-based screening in several regions (not yet in Calabria), and several other countries are at various stages of implementing this [35]. Nevertheless, as a limit of the present study, the presence of HPV-66 in the panel diagnostic tool, with the scientific evidence, could be also classified as an intermediate risk type or HSIL [36]. The reason why it has been removed from the high-risk group by the International Agency of Research in Cancer (IARC) is because HPV-66 is more prevalent in normal cytologies than in invasive cervical cancer and, therefore, it is wrong to still include it in HPV screening tests [37].

## Figures and Tables

**Table 1:** Baseline cytology grades; media age, plus or minus standard deviation; number of hrHPV genotypes; and their percentages.

Cytology Grade	Media Age (Years ± SD)	hrHPV Genotypes	Percentage (%)
NILM (n = 8)	37 ± 5.18	-	-
ASC-H (n = 24)	42 ± 8.96	**16 (3)**	**12.5**
		**18 (3)**	**12.5**
		**31 (3)**	**12.5**
		33 (2)	8.3
		39 (2)	8.3
		51 (2)	8.3
		56 (1)	4.2
		58 (1)	4.2
		66 (2)	8.3
		68 (2)	8.3
		31/58 (1)	4.2
		52/56 (1)	4.2
		59/66 (1)	4.2
CIN1 (n = 20)	40 ± 8.25	16 (1)	5
		**18 (2)**	**10**
		31 (1)	5
		**33 (2)**	**10**
		45 (1)	5
		**56 (2)**	**10**
		66 (1)	5
		68 (1)	5
		**16/18/39 (1)**	**5**
CIN2/3 (n = 12)	49 ± 10.09	16 (2)	16.6
		18 (1)	8.3
		39 (2)	16.6
		51 (1)	8.3
		56 (1)	8.3
		59 (2)	16.6
		66 (1)	8.3
		**16/18 (1)**	**8.3**
		**31/39 (1)**	**8.3**

**Table 2:** FAM19A4 and/or has-miR124-2 positivity rates, stratified per cytology grade, and their percentages.

Cytology Grade	N	n	Percentage (%)
NILM	8	0	-
ASC-H *	24	18	75%
CIN1 *	20	12	60%
CIN2/3 *	12	10	83.3%

N = group total; n = number of positive FAM19A4 and/or hsa-miR124-2 methylations. * hrHPV-positive cases include the genotypes 16, 18, 31, 33, 35, 39, 45, 51, 52, 56, 58, 59, 66, and 68.

**Table 3:** hrHPV genotyping results stratified by FAM19A4 and hsa-miR124-2 methylation positivity rates, both in combination and alone.

Gene	hrHPV Genotypes
FAM19A4 and hsa-miR124-2	**16(2)**; **18(2)**; 31(1); 33(1); 56(1); **59(2)**; 66(1)
FAM19A4	**16(4)**; **18(4)**; 31(1); 33(1); **39(3)**; 45(1); 52/56(2); 59(1); **66(2)**; 68(1)
hsa-miR124-2	**16(2)**; **18(2)**; **31(4)**; 33(1); **39(2)**; 51(1); 56(1); **58(2)**; 68(1)

**Table 4:** FAM19A4 and hsa-miR124-2 methylation positivity, in combination and alone, stratified by cytology grade and percentage.

Positivity of Methylation Genes						
	FAM19A4 and hsa-miR124-2	%	FAM19A4	%	hsa-miR124-2	%
ASC-H (n = 18)	4	22.2	6	33.4	8	44.4
CIN1 (n = 12)	3	25	6	50	3	25
CIN2/3 (n = 10)	2	20	5	50	3	30

## Data Availability

Data supporting the reported results can be found at the Unit of Microbiology and Virology, PO Pugliese, AOU Renato Dulbecco, Catanzaro, Italy.

## Supplementary Materials

The following supporting information can be downloaded at: https://www.mdpi.com/article/10.3390/pathogens13040312/s1, Figure S1: KEGG analysis in Cancer pathways. In yellow gene target for hsa-miR-124-2.

## Abbreviations

ACTB	β-actin
ASCL1	Achaete-Scute Family BHLH Transcription Factor 1
ASC-H	Atypical Squamous cells with high-grade squamous intraepithelial lesions
CADM1	Cell Adhesion Molecule 1
CESC	Cervical Squamous Cell Carcinoma and Endocervical Adenocarcinoma
CIN	cervical intraepithelial neoplasia
DPO	Dual Priming Oligonucleotide
FAM19A4	Family with Sequence Similarity 19 Member A4
GHSR	Growth Hormone Secretagogue Receptor
HPV	Human Papilloma Virus
HrHPV	High-risk Human Papilloma Virus
LHX8	LIM Homeobox 8
MAL	Myelin And Lymphocyte Protein
hsa-miR	human-microRNA
NILM	Negative for Intraepithelial Lesion Malignancy
PAX1	Paired Box 1
SST	Somatostatin
ST6GALNAC5	ST6 N-Acetylgalactosaminide Alpha-2,6-Sialyltransferase
TOCE	Tagging Oligonucleotide Cleavage and Extension
ZIC1	Zic Family Member 1
