# Supplementary material for: FAM19A4 and hsa-miR124-2 Double Methylation as Screening for ASC-H- and CIN1 HPV-Positive Women
Source: Pathogens. 2024 Apr 11;13(4):312. doi: 10.3390/pathogens13040312 (PMC11054986; doi:10.3390/pathogens13040312)
Supplement: Supplementary file 1 [file pathogens-13-00312-s001.zip › pathogens-2925372-supplementary.pdf]

pathogens

Supplementary

FAM19A4 and hsa-miR124-2 double methylation as screening for ASCUS and CIN1 HPV-positive women

Cinzia Peronace[1*§], Erika Cione[2*], Diana Marisol Abrego-Guandique[3], Marco De Fazio[1], Giuseppina Panduri[1], Maria Cristina Caroleo[3], Roberto Cannataro[4] and Pasquale Minchella[1]

[1] Unit of Microbiology and Virology, PO Pugliese, AOU Renato Dulbecco, Catanzaro, Italy; [§]current address Unit of Clinical Microbiology, PO Mater Domini, AOU Renato Dulbecco, Catanzaro, Italy cinziaperonace@hotmail.it; marco_2592@yahoo.it; pandurigiuseppina@gmail.com; pminchella@aocz.it

[2] Department of Pharmacy, Health and Nutritional Sciences, University of Calabria, 87036 Rende (CS), Italy; erika.cione@unical.it

[3] Department of Health Sciences, University of Magna Graecia, 88100 Catanzaro, Italy; dianamarisol.abregoguandique@unicz.it; mariacristina.caroleo@unicz.it

[4] Galascreen Laboratories, University of Calabria, 87036 Rende (CS), Italy; r.cannataro@gmail.com

* Equally contributed. [§]Correspondence: cinziaperonace@hotmail.it (CP); erika.cione@unical.it (EC).

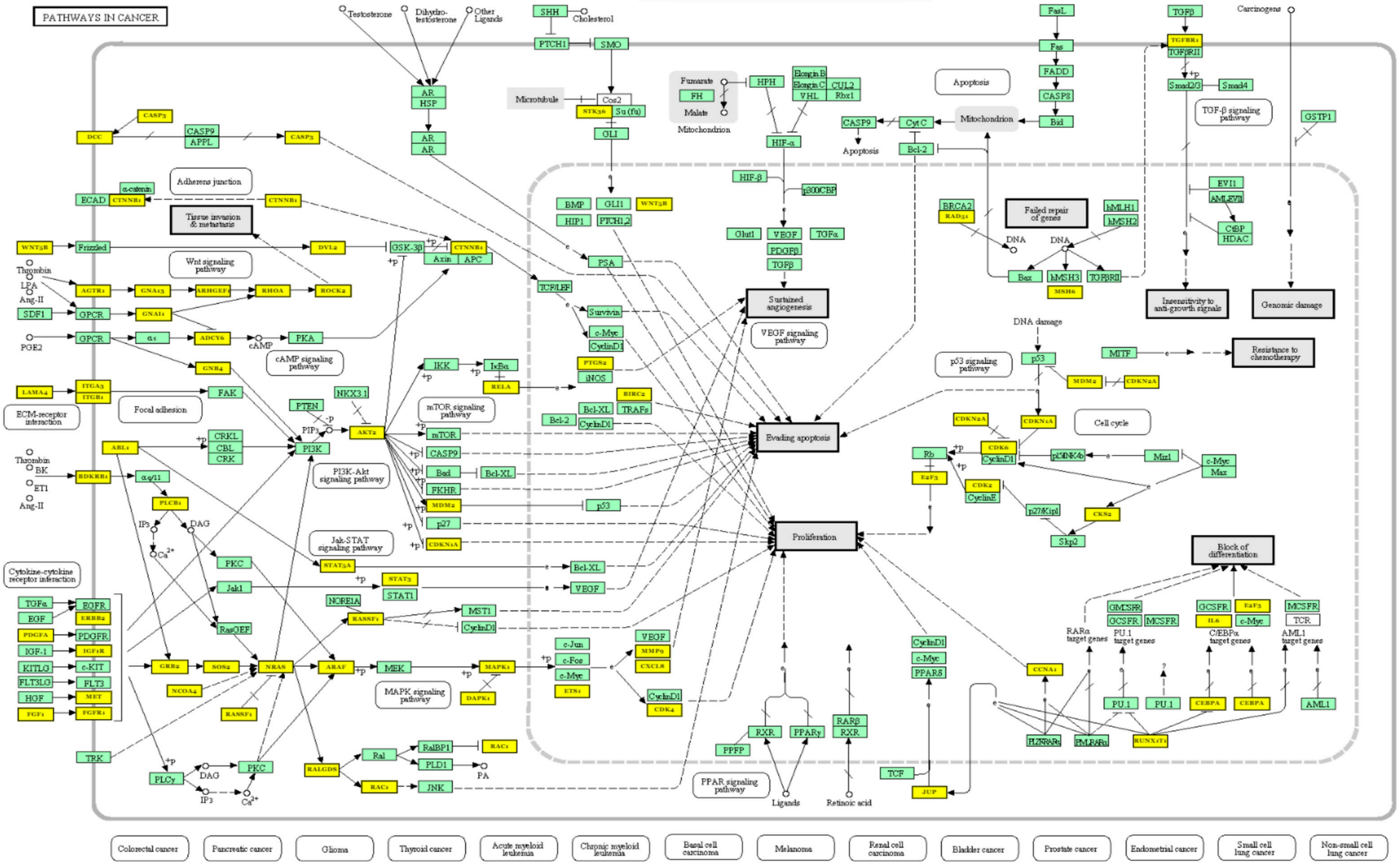

Figure S1. KEGG analysis in Cancer pathways. In yellow gene target for hsa-miR-124-2

pathogens

MDPI

Abbreviation of hsa-miR-124-2 target gene highlighted in yellow in Figure S1

ABL1: ABL Proto-Oncogene 1, Non-Receptor Tyrosine Kinase
ADCY3: Adenylate Cyclase 3
ADCY6: Adenylate Cyclase 6
ADCY9: Adenylate Cyclase 9
AGTR1: Angiotensin II Receptor Type 1
AKT2: AKT Serine/Threonine Kinase 2
ARAF: A-Raf Proto-Oncogene, Serine/Threonine Kinase
ARHGEF1: Rho Guanine Nucleotide Exchange Factor 1
BDKRB1: Bradykinin Receptor B1
BIRC2: Baculoviral IAP Repeat Containing 2
CASP3: Caspase 3
CCNA1: Cyclin A1
CDK2: Cyclin-Dependent Kinase 2
CDK4: Cyclin-Dependent Kinase 4
CDK6: Cyclin-Dependent Kinase 6
CDKN1A: Cyclin-Dependent Kinase Inhibitor 1A
CDKN2A: Cyclin-Dependent Kinase Inhibitor 2A
CEBPA: CCAAT Enhancer Binding Protein Alpha
CKS1B: CDC28 Protein Kinase Regulatory Subunit 1B
CKS2: CDC28 Protein Kinase Regulatory Subunit 2
COL4A1: Collagen Type IV Alpha 1 Chain
COL4A4: Collagen Type IV Alpha 4 Chain
CTNNB1: Catenin Beta 1
CXCL8: C-X-C Motif Chemokine Ligand 8
DAPK1: Death-Associated Protein Kinase 1
DCC: DCC Netrin 1 Receptor
DVL2: Dishevelled Segment Polarity Protein 2
E2F1: E2F Transcription Factor 1
E2F3: E2F Transcription Factor 3
ERBB2: Erb-B2 Receptor Tyrosine Kinase 2
ETS1: ETS Proto-Oncogene 1, Transcription Factor
FGF1: Fibroblast Growth Factor 1
FGF5: Fibroblast Growth Factor 5
FGFR1: Fibroblast Growth Factor Receptor 1
FN1: Fibronectin 1
GNA12: G Protein Subunit Alpha 12
GNA13: G Protein Subunit Alpha 13
GNAI1: G Protein Subunit Alpha I1
GNAI2: G Protein Subunit Alpha I2
GNAI3: G Protein Subunit Alpha I3
GNB4: G Protein Subunit Beta 4

pathogens

MDPI

GNG10: G Protein Subunit Gamma 10
GRB2: Growth Factor Receptor-Bound Protein 2
IGF1R: Insulin-Like Growth Factor 1 Receptor
IL6: Interleukin 6
ITGA3: Integrin Subunit Alpha 3
ITGB1: Integrin Subunit Beta 1
JUP: Junction Plakoglobin
LAMA1: Laminin Subunit Alpha 1
LAMA4: Laminin Subunit Alpha 4
LAMB3: Laminin Subunit Beta 3
LAMC1: Laminin Subunit Gamma 1
MAPK1: Mitogen-Activated Protein Kinase 1
MDM2: MDM2 Proto-Oncogene
MET: Mesenchymal-Epithelial Transition Factor
MMP2: Matrix Metallopeptidase 2
MMP9: Matrix Metallopeptidase 9
MSH6: MutS Homolog 6
NFKB1: Nuclear Factor Kappa B Subunit 1
NRAS: NRAS Proto-Oncogene, GTPase
PDGFA: Platelet-Derived Growth Factor Subunit A
PLCB1: Phospholipase C Beta 1
PTGS2: Prostaglandin-Endoperoxide Synthase 2
RAC1: Ras-Related C3 Botulinum Toxin Substrate 1
RAD51: RAD51 Recombinase
RALGDS: Ral Guanine Nucleotide Dissociation Stimulator
RASSF1: Ras Association Domain Family Member 1
RELA: RELA Proto-Oncogene, NF-KB Subunit
RHOA: Ras Homolog Family Member A
ROCK1: Rho-Associated Coiled-Coil Containing Protein Kinase 1
ROCK2: Rho-Associated Coiled-Coil Containing Protein Kinase 2
RUNX1T1: RUNX1 Partner Transcriptional Co-Repressor 1
SOS2: Ras/Rho Guanine Nucleotide Exchange Factor 2
STAT3: Signal Transducer and Activator of Transcription 3
STAT5A: Signal Transducer and Activator of Transcription 5A
STK36: Serine/Threonine Kinase 36
TGFBR1: Transforming Growth Factor Beta Receptor 1
WNT5B: Wingless-Type MMTV Integration Site Family, Member 5B
XIAP: X-Linked Inhibitor of Apoptosis
